# Associations of Serum 25-Hydroxyvitamin D with Physical Performance and Bone Health in Overweight and Obese Older Adults

**DOI:** 10.3390/ijerph16030509

**Published:** 2019-02-12

**Authors:** Melissa Dang, Cat Shore-Lorenti, Lachlan B. McMillan, Jakub Mesinovic, Alan Hayes, Peter R. Ebeling, David Scott

**Affiliations:** 1 Department of Medicine, Melbourne Medical School, The University of Melbourne, Parkville 3010, Australia; mdang06@gmail.com; 2 Department of Medicine, School of Clinical Sciences at Monash Health, Faculty of Medicine, Nursing and Health Sciences, Monash University, Clayton 3168, Australia; cat.shore-lorenti@monash.edu (C.S.-L.); lachlan.mcmillan@monash.edu (L.B.M.); jakub.mesinovic@monash.edu (J.M.); peter.ebeling@monash.edu (P.R.E.); 3 Australian Institute for Musculoskeletal Science (AIMSS), Department of Medicine–Western Health, Melbourne Medical School, The University of Melbourne, St Albans 3021, Australia; Alan.Hayes@vu.edu.au; 4 Institute for Health and Sport, Victoria University, Melbourne 3011, Australia

**Keywords:** 25-hydroxyvitamin D, muscle strength, physical function, bone, obesity, ageing

## Abstract

Low vitamin D status commonly accompanies obesity, and both vitamin D deficiency and obesity have been associated with falls and fracture risk in older adults. We aimed to determine the associations of serum 25-hydroxyvitamin D (25(OH)D) concentrations with physical performance and bone health in community-dwelling, overweight and obese older men and women. Serum 25(OH)D concentrations were measured in 84 participants with body mass index ≥25 kg/m^2^ (mean ± SD age 62.4 ± 7.9 years; 55% women). Physical function was determined by short physical performance battery, hand grip and quadriceps strength, and stair climb power tests. Body composition and bone structure were assessed by dual-energy X-ray absorptiometry and peripheral quantitative computed tomography, respectively. Mean ± SD 25(OH)D was 49.6 ± 17.7 nmol/L, and 50% of participants had low 25(OH)D (<50 nmol/L) levels. 25(OH)D concentrations were positively associated with quadricep strength and stair climb power in women (B = 0.15; 95% CI 0.02–0.27 kg and B = 1.07; 95% CI 0.12–2.03 W, respectively) but not in men. There were no associations between 25(OH)D and bone parameters in either sex after multivariable adjustment (all *p* > 0.05). Lower 25(OH)D concentrations are associated with poorer quadricep strength and muscle power in overweight and obese older women but not men.

## 1. Introduction

Nearly one-third of the Australian population has serum 25-hydroxyvitamin D (25(OH)D) concentrations that are considered insufficient or deficient (<50 nmol/L) [1]. Lower vitamin D has been linked with lower muscle mass and poor hand grip and quadricep strength [2,3], as well as poorer bone health [4]. This may be particularly problematic in older adults, who have increased risk of developing osteoporosis and sarcopenia and, subsequently, experiencing falls and fractures [5,6].

Within the Australian population, 29% of men and 36% of women aged 55–64 years are considered obese [7]. Obesity and low vitamin D levels are often linked together, as overweight and obese adults have a higher prevalence of vitamin D deficiency compared with normal weight adults, independent of age and geographical location [8]. Obesity may reduce 25(OH)D concentrations through sequestration into adipose tissue [9,10]. Obesity is commonly perceived to be protective against fractures in older age due to associations with higher bone mineral density (BMD), but obese older adults may have poor bone architecture and increased risk of some fracture types [11,12]. Obesity also leads to poor physical performance, as demonstrated through impaired balance, slower gait speeds and weaker relative lower limb strength [13,14], and obese older adults also have increased fall risk [11,15].

Despite these known associations, few studies have investigated the effects of low vitamin D on musculoskeletal health in obese older adults. In middle-aged adults (mean age 44 years) with severe obesity (body mass index (BMI) ≥40 kg/m^2^), a negative association was observed between serum 25(OH)D levels and walking time measured over 500 m [16]. Two separate studies found no significant associations between vitamin D and bone health in middle-aged and older overweight and obese adults [17,18]. Further research is required to clarify the associations of 25(OH)D with musculoskeletal outcomes in the overweight and obese older adult population. We aimed to investigate the associations of serum 25(OH)D with physical performance and bone health in a cross-sectional study of overweight and obese community-dwelling older adults.

## 2. Materials and Methods

### 2.1. Study Design and Participants

This is a secondary analysis of a cross-sectional study of 84 community-dwelling overweight or obese (BMI ≥ 25 kg/m^2^) adults aged 50 years and older [19]. Exclusion criteria included vitamin D supplementation exceeding 400 IU/day, contraindications to musculoskeletal imaging and medical conditions that would impede physical performance assessments, such as neurological conditions.

The participants were recruited via print and online advertising. All the assessments were completed between 2014 and 2016, across all the seasons of the year, at Sunshine Hospital. Ethics approval was provided by the Melbourne Health Human Research Ethics Committee (reference number: 2013.294). The participants provided written informed consent prior to enrolment, and all aspects of the study complied with the World Medical Association Declaration of Helsinki–Ethical Principles for Medical Research Involving Human Subjects.

### 2.2. Anthropometrics

Weight (Seca 804 electronic scales, Seca, Hamburg, Germany) and height (Seca 222, wall-mounted stadiometer, Seca, Hamburg, Germany) were measured with footwear and any heavy items of clothing removed. BMI was calculated using the formula: weight (kg)/(height (m)^2^). All anthropometric measurements were recorded twice and then averaged.

### 2.3. Questionnaire

A self-administered questionnaire assessed patient demographics and medical history. Weekly amount of physical activity was determined using the Active Australia Survey [20].

### 2.4. Blood Biochemistry

Blood samples were collected following an overnight fast by an experienced phlebotomist at Dorevitch Pathology, Sunshine Hospital. The sample was analysed for serum 25(OH)D concentrations via a DiaSorin LIAISON (DiaSorin Inc., Stillwater, MN, USA) chemiluminescent immunoassay. The assay has a dynamic range from 10 nmol/L to 375 nmol/L and a coefficient of variation (CV) of <10% [21]. Vitamin D deficiency was defined as a serum level less than 50 nmol/L [22].

### 2.5. Muscle Strength and Physical Function

Hand grip strength was assessed in both hands using a Jamar Plus Digital hydraulic hand grip dynamometer (Patterson Medical, Bolingbrook, IL, USA). The dynamometer was held with the elbow flexed at a 90° angle while the participant was seated. Hand grip strength was assessed 3 times with an average taken from the latter 2 tests of both limbs.

Quadricep strength was measured in both legs with a hand-held dynamometer (HHD; Lafayette Manual Muscle Tester Model 01165, Lafayette Instrument, IN, USA) while the participants were seated with hips and knees at 90° flexion, feet just above the floor and hands resting in their lap. The HHD was placed 10 cm above the ankle joint. The participants were instructed to slowly apply maximal force to the dynamometer and peak force was recorded. The mean of the latter 2 of 3 readings was later calculated.

Stair climb power was used to assess lower limb power and, therefore, mobility performance and risk of disability [23]. The participants were timed as they climbed a 10-step flight of stairs as quickly as possible. Handrails were allowed to be used, if required. The mean of 2 tests was taken. Power was calculated using the formula: power = force × velocity. Velocity was calculated with the vertical distance of the stairs (1.73 m) divided by the time it took the participant to ascend. Force was measured with the mass of the participant multiplied by acceleration due to gravity (9.8 m/s^2^).

The short physical performance battery (SPPB), consisting of three tasks (repeated chair stands, standing balance and gait speed test over a distance of 2.44 m), is a highly validated measure of physical performance and disability in the elderly [24] and was performed by all the participants. A score from 0 to 12 is generated from the 3 tests, with a higher score indicating better function. A score of 9 or less is considered indicative of poor physical performance [25].

### 2.6. Body Composition and Bone Health

Whole-body, dual-energy X-ray absorptiometry (DXA; Hologic Discovery W, Hologic, Bedford, MA, USA) was used to determine total body fat percentage, appendicular lean mass and total body BMD (minus head). The device was calibrated on each study day using a spine phantom. Peripheral quantitative computed tomography (pQCT; Stratec XCT3000, Stratec Medizintechnik GmbH, Pforzheim, Germany) scans, with 2.5-mm thickness, a voxel size of 0.8 mm and scan speed of 20 mm/s, were taken of each participant’s non-dominant forearm and lower leg. All the images were analysed with the manufacturer’s software (version 6.2, Pforzheim, Germany). However, 2 scans were removed from analysis due to movement artefacts. The scans were taken at 66% (diaphyseal) and 4% (distal) of the radial and tibial length, measuring proximally from the distal radioulnar joint to the olecranon and tibiotarsal joint and tibial plateau, respectively. Total, cortical and trabecular density, area and thickness measurements were obtained from these sites. Trabecular bone was identified from the distal tibia and radius, using the manufacturer’s default setting, which concentrically separates the outer 55% of the bone area and defines the inner 45% as trabecular bone. Cortical bone was identified at the 66% site of the tibia and radius using the default threshold of 710 mg/cm^3^ [26]. The device was calibrated daily using the manufacturer’s phantom. The CV for phantom density was 0.2% for the duration of the study.

### 2.7. Statistical Analysis

All data analysis was completed using SPSS Statistics Version 23 (SPSS Inc., Chicago, IL, USA). Continuous variables were assessed for normality, and non-parametric tests were used where appropriate. One-way ANOVA compared 25(OH)D levels across the season of blood collection. The participants were stratified by vitamin D status (25(OH)D < 50 nmol/L vs. ≥ 50 nmol/L). Independent samples t-tests, Mann–Whitney U tests and Chi-square tests were performed to compare sex-specific characteristics between the 25(OH)D groups. An a priori decision was made to stratify analyses by sex given the evidence of sex-specific associations between 25(OH)D levels and muscle and bone health [27,28,29]. Nevertheless, evidence of an interaction between vitamin D status and sex for physical function was observed in this cohort; indeed, a significant interaction between these variables was observed for the outcome of stair climb power (interaction term *p* = 0.041). Pearson and Spearman correlations were used to determine associations between 25(OH)D concentrations and muscle and bone outcomes in all the participants. Sex-stratified multivariable linear regression analyses determined whether associations of 25(OH)D with bone and muscle outcomes were significant after adjustment for potential confounders. Separate regression models were fitted for each musculoskeletal outcome as a dependent variable, with 25(OH)D levels and other covariates included as independent variables. Model 1 was adjusted for age, and Model 2 was adjusted for age, duration of weekly moderate and vigorous physical activity and total body fat percentage. P-values of less than 0.05 or 95% confidence intervals (CI) that did not include the null point were considered statistically significant.

## 3. Results

Approximately 48% of the 84 study participants had a BMI between 25 and 30 kg/m^2^ (overweight), and the remainder were classified as obese (BMI ≥ 30 kg/m^2^). The majority (58%) of the participants had measurements collected during winter, but there were no associations between 25(OH)D levels and season of blood collection (*p* = 0.266). Only 6 participants reported use of vitamin D supplements; 2 had serum 25(OH)D ≥ 50 nmol/L, while 4 had 25(OH)D < 50 nmol/L. Table 1 reports descriptive characteristics according to serum 25(OH)D status and sex. A total of 50% of participants had a serum 25(OH)D concentration lower than 50 nmol/L. There was an equal number of women and men in each of their respective 25(OH)D status groups. Men with 25(OH)D ≥ 50 nmol/L were significantly older and had lower stair climb power than men with 25(OH)D < 50 nmol/L. More women with 25(OH)D < 50 nmol/L had self-reported diabetes than women with 25(OH)D ≥ 50 nmol/L. No other differences were identified between 25(OH)D groups in men or women.

In univariate analyses, there was a positive correlation between 25(OH)D and age in men but not women, whereas serum 25(OH)D concentrations were positively correlated with weekly physical activity and quadricep strength in women only (Table 2). There were no other significant correlations between 25(OH)D, body composition or physical function in either sex.

There was a positive correlation between serum 25(OH)D concentrations and total bone area of the distal radius in men (Table 3). There was also a negative correlation between serum 25(OH)D concentrations and trabecular density of the distal tibia in women. No other significant correlations were observed between 25(OH)D and bone parameters.

Table 4 presents the results of sex-stratified multivariable linear regression analyses of associations of serum 25(OH)D with physical performance and bone parameters. Model 1, which was adjusted for age, demonstrated that 25(OH)D concentrations were positively associated with quadricep strength in women. This remained significant in Model 2 after further adjustment for physical activity and total body fat percentage. Stair climb power was also significantly positively associated with 25(OH)D concentrations in women in Model 2. Negative associations of 25(OH)D with total and trabecular densities at the distal tibia were significant for women in Model 1 but not in the fully adjusted model. In the fully adjusted model for men, 25(OH)D was negatively associated with gait speed. A positive association was also observed in men between 25(OH)D and total area of the distal radius in Model 1 but not Model 2.

## 4. Discussion

This cross-sectional study of community-dwelling, overweight and obese older adults demonstrated that serum 25(OH)D concentrations are positively associated with quadricep strength and stair climb power in women, but negatively associated with gait speed in men. No associations between 25(OH)D and bone parameters were identified in men or women after multivariable adjustment for age, self-reported weekly physical activity and total fat percentage. Nevertheless, given the observed associations with weaker quadricep strength and poorer lower limb power, overweight and obese older women with low vitamin D concentrations may be at a higher risk for future falls and fall-related injury [30,31]. Longitudinal studies in large cohorts are required to determine associations of 25(OH)D with incidence of falls and fractures in overweight and obese older women.

Low vitamin D concentrations were associated with a decrease in lower limb strength and muscle quality in older men and women in a longitudinal study [3]. A trial also observed an 8% increase in quadricep strength in both older men and women after one year of vitamin D supplementation [32]. We have expanded on the existing literature by demonstrating that in the overweight and obese older population, an association of lower vitamin D concentrations with weaker quadriceps strength is observed in women but not men. This sex-specific phenomenon may be explained by the fact that particular polymorphisms of the vitamin D receptor (VDR) gene have been reported to predispose elderly women to lower limb weakness [33]. Older women with the homozygous dominant (BB) VDR genotype for the Bsm1 gene have significantly weaker quadricep strength compared with women with the heterozygous (Bb) or recessive homozygous (bb) genotypes. In addition, the absence of the BB genotype is also associated with increased body weight and fat mass, suggesting that variations in VDR polymorphisms in women may predispose them to both poorer lower limb strength and obesity [34]. In contrast, elderly men with the genotype BB have significantly greater knee extensor strength compared with those with the Bb/bb genotype [35] and have higher BMIs and waist circumference [36]. However, given that no genotyping was completed in this study, it is not possible to determine that the sex-specific associations that we observed are due to variations in genotypes. Indeed, another contributing factor may be that the women within our cohort reported significantly lower physical activity compared with men (*p* = 0.009; data not shown), and it is possible that men who are overweight or obese maintain higher physical activity levels than their female counterparts and that this mitigates the potential negative effects of low vitamin D on physical function in overweight or obese men [37]. Whilst vitamin D is primarily stored in adipose tissue, there is evidence of vitamin D uptake in skeletal muscle [38]. Indeed, higher physical activity may stimulate osteoblast function through the mobilisation of vitamin D in skeletal muscle stores [39]. It is therefore possible that the higher muscle mass of overweight and obese men relative to women also influences the relationship between vitamin D status and muscle function. It is notable that in the present study, a correlation of approximately −0.2 was observed between 25(OH)D and BMI in both men and women. While this was non-significant, it does suggest a weak correlation between vitamin D status and body size, consistent with the current understanding [8]. Nevertheless, no associations were observed for total body fat or appendicular lean mass with 25(OH)D, and so, further research is required to determine how body composition influences vitamin D status in overweight and obese older adults. 

Few studies have specifically investigated the effects of vitamin D on musculoskeletal health in overweight and obese individuals. A cross-sectional study conducted by Ahern et al. reported slower walking times in 252 severely obese and vitamin D deficient adults [16]. Our study differs from that study in both inclusion criteria and methodology. We recruited an older population (mean age ± SD 62.4 ± 7.9 years vs. 43.7 ± 11.2 years) and examined various predictors of falls and fracture, such as quadriceps strength and stair climb power, as well as bone parameters. To our knowledge, our study is the first to identify an association between low vitamin D levels and lower quadricep strength and stair climb power in overweight and obese older women.

However, somewhat surprisingly, a negative association was observed between 25(OH)D and gait speed in men. This association was unexpected, although vitamin D has been described to have a U-shaped relationship with physical function, with plasma 25(OH)D levels greater than 120 nmol/L associated with a poorer performance on the sit-to-stand test [40]. While we did not observe significant non-linear associations between 25(OH)D concentrations and physical performance measures in the present study (all *p* > 0.05; data not shown), high-dose vitamin D supplementation (single dose of 500,000 IU per year for 3–5 years) has also been linked to an increased risk of falls and fracture [41], potentially mediated through a decrease in hip flexion strength [42]. A recent study in postmenopausal women who had mean ± SD of serum 25(OH)D concentrations of 90 ± 14 nmol/L following vitamin D supplementation (2800 IU/day) demonstrated declines in physical performance compared with the placebo group [43]. While the men in our study did not demonstrate 25(OH)D levels consistent with high-dose supplementation, our results suggest the possibility that the optimal 25(OH)D levels for maintaining physical function may differ between overweight and obese men and women. The recruitment of specifically overweight and obese adults may also explain this result, as it has been well documented that obesity is associated with slower gait speeds [14], and optimal levels of 25(OH)D for physical function may also be influenced by obesity status. Ageing is associated with slower gait speeds, and the fact that the men with higher 25(OH)D concentrations were surprisingly significantly older than those with lower concentrations may also have contributed to this association [44]; while we adjusted for age in this analysis, it is possible that other age-related conditions make a stronger contribution to gait speed than 25(OH)D concentrations.

Despite its known role in bone metabolism, the effect of low 25(OH)D concentrations on bone structure has not been consistently demonstrated in radiological studies [45]. A meta-analysis concluded that supplemental vitamin D monotherapy was only effective in improving BMD in the femoral neck [46], which is a site that was not assessed in the present study. Regardless, the lack of significant association with structural bone parameters at the radius in our study is consistent with the minimal changes in BMD of the forearm found on DXA in this meta-analysis [46]. Our study also partially confirms the findings of a study with similar participant characteristics and methodology, in which it was reported that obesity is not associated with BMD loss at the tibia and radius and that low 25(OH)D levels do not lead to BMD loss [18]. Overweight and obese men in the present study exhibited a significant positive correlation between 25(OH)D and the total area of the distal radius, but this was not significant in the fully adjusted model. However, this may be due to the low statistical power of the study. A more recent study of 633 adults that were normal weight, overweight and obese reported sex-specific associations in bone parameters. In particular, obese women exhibited a weak negative correlation between serum 25(OH)D and distal radius trabecular density and tibial shaft cortical strength index [17]. In age-adjusted analyses in the present study, 25(OH)D was similarly negatively associated with distal tibial total and trabecular density in women, but these associations were non-significant after further adjustment. This may be due to the fact that bone structure depends on the interactions of multiple other factors, which were not assessed in this study. For example, parathyroid hormone (PTH) and calcium in diet has been more strongly associated with bone health than 25(OH)D in overweight and obese older adults [47]. It is also possible that obesity itself, through mechanical loading, may counterbalance the deleterious effects low vitamin D levels have on bone structure. However, we believe this to be unlikely, as obesity appears to be protective of axial skeleton fractures [48] but leads to cortical bone volumetric BMD loss at distal sites, such as the radius and tibia [11]. This is an area that needs further clarification as the majority of studies looking at the role of low vitamin D levels on bone health have primarily done so through the measurement of BMD via DXA. The use of pQCT and high-resolution peripheral quantitative computed tomography (HR-pQCT) in future studies may clarify the effects of vitamin D on bone structure and geometry in overweight and obese adults [49].

There are several limitations to this study. Firstly, the causality of the associations identified in this study cannot be determined as a consequence of its cross-sectional design. The study population consisted of individuals recruited by convenience sampling and was relatively small; post-hoc analyses indicate that we had 90 and 95% power (alpha = 0.05) to detect moderate correlations between study variables in men and women, respectively, but these sample sizes may have been insufficient to detect weaker associations. Our sample size was also reduced by the a priori decision to stratify analyses by sex. We felt this decision was justified given the evidence of sex-specific associations from previous studies [27,28,29] and the interaction demonstrated between vitamin D status and sex for stair climb power in our analysis, but future studies are needed to confirm these sex-specific associations. While the musculoskeletal outcomes assessed in this study have been associated with falls and fracture risk, longitudinal studies with large sample sizes will be required to assess the effects of vitamin D on the incidence of falls and fractures in overweight and obese older adults. We also did not collect data on the prevalence of falls and fractures, and physical activity was self-reported, which may be a source of bias. Measurements of PTH, phospho-calcium balance and renal function were not included in our study and may provide clarity on the relationship between vitamin D and musculoskeletal health given known influences on vitamin D, calcium and bone mineral homeostasis. VDR genotypes were also not measured in this study, and therefore, we are uncertain if the observations were due to variations in genotypes. Lastly, the study was focused on participants who were overweight and obese community-dwelling, and therefore, the results may not be generalisable to older severely obese populations or to adults who are institutionalised. Nevertheless, the results from this study highlight that overweight and obese older women in particular may benefit from higher vitamin D levels for the prevention of functional decline and, potentially, to decrease their risk of future falls and fracture.

## 5. Conclusions

Lower 25(OH)D levels are associated with lower quadriceps strength and muscle power in overweight and obese older women but not men. There were no associations between serum 25(OH)D and bone parameters for either sex after multivariable adjustment. Further research is required to confirm these associations and to determine whether the correction of vitamin D deficiency can improve physical function and reduce the risk of falls in overweight and obese women.

## Figures and Tables

**Table 1:** Sex-specific comparisons for baseline characteristics between 25(OH)D groups.

Characteristics	Women		*p*-Value	Men		*p*-Value
	25(OH)D ≥ 50 N = 23	25(OH)D < 50 N = 23		25(OH)D ≥ 50 N = 19	25(OH)D < 50 N = 19	
**Age (years)**	60.7 ± 6.6	63.3 ± 8.5	0.253	65.8 ± 8.6	59.8 ± 7.0	**0.024**
**BMI (kg/m^2^)**	33.0 ± 7.7	33.5 ± 5.2	0.823	30.6 ± 5.5	32.3 ± 5.4	0.338
**Total body fat percentage (%)**	42.1 ± 6.2	43.2 ± 4.9	0.499	29.5 ± 6.2	30.3 ± 4.9	0.672
**Self-reported diabetes (%) ^#^**	8.7	34.8	**0.032**	10.5	31.6	0.111
Moderate and vigorous physical activity (min/week) *	45.0 (0.0, 180.0)	2.0 (0.0, 40.0)	0.152	120.0 (0.0, 360.0)	120.0 (0.0, 300.0)	0.906
**Time spent outdoor (hours/week)**	4.3 ± 3.4	4.6 ± 3.0	0.782	7.6 ± 5.6	6.7 ± 4.5	0.560
**Serum 25(OH)D (nmol/L)**	62 ± 11	36 ± 10	**<0.001**	66 ± 11	35 ± 10	**<0.001**
**Hand grip strength (kg)**	22.9 ± 5.9	23.4 ± 5.9	0.810	37.6 ± 7.5	38.9 ± 7.3	0.607
**Quadricep strength (kg)**	15.7 ± 6.9	12.4 ± 6.5	0.106	22.1 ± 9.8	19.6 ± 11.0	0.467
**Gait speed (cm/s)**	80.6 ± 20.2	76.1 ± 17.6	0.435	74.1 ± 18.4	79.5 ± 11.2	0.285
**Stair climb power (W)**	254.0 ± 84.0	244.0 ± 59.9	0.646	283.2 ± 83.1	337.9 ± 66.1	**0.031**
**SPPB score**	10.0 ± 1.9	9.8 ± 1.7	0.618	9.8 ± 2.2	9.7 ± 1.4	0.860
**66% site of tibia cortical density (mg/cm^3^)**	1042.90 ± 48.51	1040.48 ± 32.85	0.844	1050.65 ± 32.67	1064.25 ± 38.66	0.250
**Distal tibia trabecular density (mg/cm^3^)**	222.58 ± 49.75	234.15 ± 40.69	0.406	240.02 ± 37.99	250.13 ± 35.08	0.400
**66% site of radius cortical density (mg/cm^3^)**	1074.85 ± 54.90	1059.49 ± 62.53	0.381	1070.17 ± 53.63	1085.44 ± 45.83	0.352
**Distal radius trabecular density (mg/cm^3^)**	171.28 ± 31.62	173.29 ± 37.23	0.845	204.03 ± 41.21	214.11 ± 35.67	0.425

All data are mean ± SD unless otherwise specified. Abbreviations: 25(OH)D: 25-hydroxycholcalciferol; BMI: body mass index; and SPPB: short physical performance battery. ^#^ Proportions compared using Chi-square tests; * median, inter-quartile range (IQR) compared using Mann–Whitney U tests. Bold *p*-values are significant.

**Table 2:** Correlation coefficients (*p*-values) for associations of 25(OH)D with age, body composition, physical activity and muscle parameters.

Characteristic	Serum 25(OH)D (nmol/L)	
	Women	Men
Age (year) *	0.005 (0.976)	**0.373 (0.021)**
BMI (kg/m^2^) *	−0.202 (0.178)	−0.213 (0.199)
Total body fat percentage (%) *	−0.063 (0.679)	−0.183 (0.272)
Appendicular lean mass (kg) *	−0.093 (0.538)	−0.076 (0.652)
Physical activity (min/week) *	**0.302 (0.042)**	0.098 (0.557)
Time spent outdoors (hours/week) *	0.053 (0.725)	0.083 (0.621)
Hand grip strength (kg) *	−0.008 (0.956)	0.061 (0.717)
Quadricep strength (kg) *	**0.334 (0.023)**	0.174 (0.295)
**Gait speed (m/s)**	0.165 (0.272)	−0.225 (0.175)
Chair standing time (s) *	0.011 (0.941)	−0.171 (0.311)
**Stair climb power (W)**	0.124 (0.412)	−0.254 (0.124)
SPPB score *	0.069 (0.647)	0.087 (0.605)

All correlations are Pearson correlation coefficients unless otherwise specified. Abbreviations: 25(OH)D: 25-hydroxycholcalciferol; BMI: body mass index; and SPPB: short physical performance battery. * Spearman correlations. Bold *p*-values are significant.

**Table 3:** Correlation coefficients (P-values) for associations of 25(OH)D with bone parameters.

Characteristic	Serum 25(OH)D (nmol/L)	
	Women	Men
Distal tibia total density (mg/cm^3^) *	−0.264 (0.083)	−0.095 (0.569)
**Distal tibia total area (mm^2^)**	0.030 (0.846)	0.151 (0.364)
**Distal tibia trabecular density (mg/cm^3^)**	**−0.307 (0.043)**	−0.165 (0.321)
**66% site of tibia cortical density (mg/cm^3^)**	0.163 (0.280)	−0.050 (0.764)
**66% site of tibia cortical area (mm^2^)**	0.048 (0.750)	0.047 (0.779)
**66% site of tibia cortical thickness (mm)**	0.020 (0.895)	0.056 (0.737)
**Distal radius total density (mg/cm^3^)**	−0.108 (0.474)	−0.082 (0.626)
Distal radius total area (mm^2^) *	−0.038 (0.801)	**0.422 (0.008)**
Distal radius trabecular density (mg/cm^3^) *	−0.131 (0.384)	−0.060 (0.720)
**66% site of radius cortical density (mg/cm^3^)**	0.103 (0.497)	−0.086 (0.608)
**66% site of radius cortical area (mm^2^)**	−0.008 (0.958)	−0.001 (0.996)
**66% site of radius cortical thickness (mm)**	−0.090 (0.553)	−0.244 (0.141)
Total body BMD (mg/cm^2^) *	−0.255 (0.088)	0.011 (0.949)

All correlations are Pearson correlation coefficients unless otherwise specified. Abbreviations: 25(OH)D: 25-hydroxycholcalciferol; and BMD: bone mineral density. * Spearman correlations. Bold *p*-values are significant.

**Table 4:** Sex-specific multivariable linear regression analyses (unstandardized B-coefficient (95% CI)) exploring associations between serum 25(OH)D and musculoskeletal outcomes categorised by sex.

Characteristic	Model 1 Adjusted for Age		Model 2 Adjusted for Age, Physical Activity and Total Fat Percentage	
	Women	Men	Women	Men
**Hand grip strength (kg)**	0.007 (−0.095, 0.110)	0.108 (−0.013, 0.228)	0.022 (−0.075, 0.119)	0.110 (−0.014, 0.234)
**Quadricep strength (kg)**	**0.134 ** **(0.017, 0.251)**	0.112 (−0.085, 0.308)	**0.149 ** **(0.024, 0.274)**	0.096 (−0.102, 0.293)
**Gait speed (cm/s)**	0.183 (−0.157, 0.523)	−0.225 (−0.508, 0.058)	0.236 (−0.125, 0.597)	**−0.272** **(−0.528, −0.016)**
**Chair standing time (s)**	−0.020 (−0.209, 0.169)	0.013 (−0.105, 0.131)	−0.025 (−0.224, 0.174)	0.039 (−0.073, 0.151)
**Stair climb power (W)**	0.474 (−0.598, 1.546)	−0.854 (−2.309, 0.600)	**1.074 ** **(0.115, 2.033)**	−0.984 (−2.413, 0.446)
**SPPB score**	0.004 (−0.028, 0.036)	0.005 (−0.029, 0.039)	0.012 (−0.022, 0.045)	0.000 (−0.034, 0.034)
**Distal tibia total density (mg/cm^3^)**	**−0.763** **(−1.466, −0.061)**	−0.088 (−0.742, 0.567)	−0.693 (−1.467, 0.082)	−0.087 (−0.768, 0.594)
**Distal tibia total area (mm^2^)**	0.274 (−2.562, 3.109)	−0.230 (−2.933, 2.473)	0.304 (−2.370, 2.978)	−0.269 (−3.064, 2.525)
**Distal tibia trabecular density (mg/cm^3^)**	**−0.843** **(−1.640, −0.046)**	−0.335 (−1.026, 0.355)	−0.827 (−1.708, 0.053)	−0.331 (−1.049, 0.388)
**66% site of tibia cortical density (mg/cm^3^)**	0.376 (−0.316, 1.069)	0.025 (−0.654, 0.704)	0.510 (−0.211, 1.231)	0.027 (−0.675, 0.728)
**66% site of tibia cortical area (mm^2^)**	0.097 (−0.607, 0.800)	0.148 (−0.790, 1.085)	0.174 (−0.577, 0.924)	0.122 (−0.852, 1.096)
**66% site of tibia cortical thickness (mm)**	0.000 (−0.010, 0.011)	0.003 (−0.010, 0.017)	0.002 (−0.009, 0.013)	0.003 (−0.011, 0.017)
**Distal radius total density (mg/cm^3^)**	−0.350 (−1.174, 0.474)	−0.231 (−1.318, 0.855)	−0.405 (−1.285, 0.475)	−0.228 (−1.361, 0.905)
**Distal radial total area (mm^2^)**	−0.003 (−1.024, 1.018)	**1.546** **(0.042, 3.049)**	0.165 (−0.841, 1.172)	1.493 (−0.060, 3.046)
**Distal radius trabecular density (mg/cm^3^)**	−0.364 (−0.954, 0.226)	−0.029 (−0.767, 0.708)	−0.361 (−0.998, 0.277)	−0.063 (−0.819, 0.693)
**66% site of radius cortical density (mg/cm^3^)**	0.324 (−0.650, 1.298)	0.080 (−0.819, 0.980)	0.339 (−0.708, 1.387)	0.040 (−0.891, 0.970)
**66% site of radius cortical area (mm^2^)**	−0.011 (−0.202, 0.179)	−0.007 (−0.253, 0.239)	−0.005 (−0.207, 0.197)	−0.004 (−0.260, 0.253)
**66% site of radius cortical thickness (mm)**	−0.002 (−0.009, 0.005)	−0.004 (−0.010, 0.003)	−0.002 (−0.010, 0.005)	−0.004 (−0.010, 0.002)
**Appendicular lean mass (kg)**	−0.005 (−0.091, 0.080)	0.010 (−0.054, 0.074)	0.016 (−0.069, 0.101)	0.018 (−0.045, 0.082)
**Total body BMD (mg/cm^2^)**	−2.093 (−4.59, 0.404)	−0.089 (−2.949, 2.771)	−1.567 (−4.206, 1.072)	0.070 (−2.881. 3.022)

Abbreviations: 25(OH)D: 25-hydroxycholcalciferol; BMI: body mass index; SPPB: short physical performance battery; and BMD: bone mineral density. Bold *p*-values are significant.
